# Long segment Rosai-Dorfman disease-causing spinal cord compression: A case report

**DOI:** 10.1016/j.ijscr.2022.106775

**Published:** 2022-01-15

**Authors:** Rabiul Karim, Mohammad Majed Sultan, Kamal Hossain, Himel Chowdhury, Moshiur Rahman

**Affiliations:** a Neurosurgery Department, Chittagong Medical College Hospital; b Neurosurgery Department, Holy Family Red Crescent Medical College, Dhaka, Bangladesh

**Keywords:** Rosai-Dorfman disease, Sinus histiocytosis with massive lymphadenopathy, MRI, Spinal extradural lesion, Long segment, Laminoplasty

## Abstract

**Introduction and importance:**

Rosai-Dorfman disease is a rare, histiocytic lymphoproliferative disease of unknown etiology. It manifests mainly as painless cervical lymphadenopathy, with very few cases reported extranodal involvement in the central nervous system. Isolated spinal Rosai-Dorfman disease is sporadic.

**Case presentation:**

This case report documents a rare instance of an isolated long-segment spinal Rosai-Dorfman disease (C4-D6) along with the review of relevant literature. A 33-year male presented with progressive quadriparesis and urinary retention. A magnetic resonance scan (MRI) revealed a long segment epidural lesion from C4-D6 levels that led to the displacement of the cord. A core biopsy of the spinal tumor revealed characteristic histiocytic emperipolesis and confirmational immunocytohistochemistry markers, confirming the diagnosis. Surgical resection and laminoplasty were performed.

**Clinical discussion:**

The following histopathology and immunocytohistochemistry findings showed the presence of histiocytes positive for S100 and CD68 positive. Therefore, it was diagnosed to be a case of Rosai-Dorfman disease. The patient had a smooth postoperative recovery and displayed marked motor improvement in the ensuing days. This is a rare case that posed an intriguing challenge to approach.

**Conclusion:**

To our knowledge, we have encountered one of the most prolonged segmental lesions in isolated spinal Rosai-Dorfman diseases, where surgical management (surgical resection and laminoplasty) has proven to bring about remarkable improvement.

## Highlights

• Rosai-Dorfman disease is a rare, histiocytic lymphoproliferative disease.
• Isolated spinal Rosai-Dorfman disease is sporadic.
• Histological examinations and immunophenotyping are used for diagnosis.

## 1 Introduction

Rosai-Dorfman Disease (RDD) is a rare, benign, self-limiting lymphoproliferative illness with an unknown cause initially identified in 1969 by Juan Rosai and Ronald Dorfman [1]. It's also known as sinus histiocytosis with extensive lymphadenopathy, and it's characterized by an overabundance of histiocytes in the body's lymph nodes, with cervical lymph node involvement being the most common [2]. The disease mainly affects the lymph nodes, with large, painless cervical lymphadenopathy as its hallmark.

Fever, lethargy, weight loss, and anemia are other common symptoms [3]. Extranodal involvement is uncommon, occurring in just 30–40% of RDD cases, and typically affects the skin, upper respiratory tract, eye and orbit, salivary gland, testes, and bone [4]. The involvement of the central nervous system is extremely rare, accounting for only 5% of all cases of extranodal illness. Even rarer is isolated spinal RDD, which accounts for around 20–25% of all RDD patients with CNS disease [5] and is usually not accompanied by lymphadenopathy [6]. Children and young people are the most common victims of spinal RDD, with a slight male preponderance [3].

In neurosurgery, RDD is a fascinating and still unknown clinical condition. This case report includes a synopsis of the clinical presentation of a unique case of isolated spinal Rosai-Dorfman illness, including a lengthy spinal segment causing extradural cord compression. This case report followed the SCARE guidelines for its realization [7].

## 2 Case report

A 33-year male presented to the Department of Neurosurgery, Chittagong Medical College and Hospital, in November 2019, with a 20-day history of insidious onset and gradually progressive bilateral lower limb weakness accompanied by weak hand grips. He also complained of urinary retention for seven days. In addition, the patient reported mild back pain in the upper dorsal region, which was exacerbated at night and relieved upon intake of analgesics. The patient had an average build with no other associated abnormalities on general examination. Neurological examination revealed muscle wasting and hypertonia of both lower limbs. His motor power was marked reduced (Grade 1) in both lower limbs. There were exaggerated deep tendon reflexes and sustained bilateral ankle clonus. The plantar response was extensor bilaterally. All modalities of sensation were diminished below the D10 level. Examination of upper extremities showed intact muscle strength (Grade 5) for all groups of muscles except in the grips of the hands (Grade 1). Tricep jerks were absent bilaterally. Hoffman's sign was missing. There was a catheter in situ for urinary retention.

## 3 Intervention

Excision of the epidural lesion was performed from C4 to D6 levels. The lesion was grey-white, firm in consistency, and adherent to the dura mater, ligament, and laminae. Nerve roots were free from the lesion. Laminoplasty was done throughout its entire length (C4 to D6). The following preoperative and postoperative images illustrate the procedure:

The dissected spinal tumor was placed, and a sample segment was sent for histopathological examination.

A long-segment epidural lesion of the spine, approximately 11 cm in length after excision from C4-D6. After that, an epidural specimen measuring 2 × 1.5 × 1 cm consisting of an irregular, grey-white, solid cut piece of tumor was sent for histopathological examination. The section revealed massive, spherical, vesicular nuclei with a fragile nuclear membrane, conspicuous nucleoli, and enormous histiocytes harboring intact lymphocytes (emperipolesis, lymphocytophagocytosis). Atypia and multinucleation are two characteristics of histiocytes. Lymphocytes and polyclonal plasma cells can be detected. Histocytes expressing S100 and CD68 proteins were discovered by immunocytohistochemistry. As a result, a Rosai-Dorfman Disease working diagnosis has been established.

## 4 Prognosis

The patient had a smooth postoperative (Fig. 2) recovery and exhibited marked improvement in the motor power of both his upper and lower limbs, evidenced by his ability to walk (with support) steadily. Postoperative imaging showed the patient had also completely regained control over urine and stool.

**Fig. 1:**
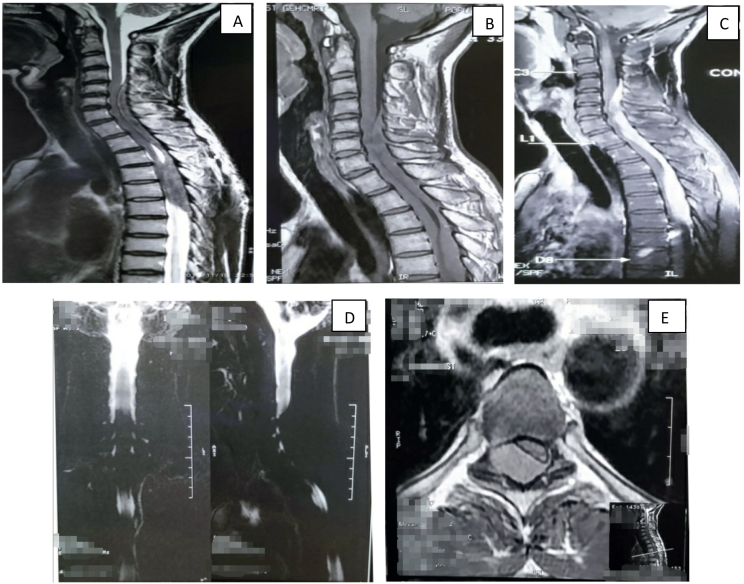
(A-E): MRI of the whole spine revealed a large elongated epidural mass in the posterior aspect of the spinal canal extending from C4-D6 levels resulting in flattening of the spinal cord, anterior displacement of line, and cord compression (Fig. 1). Enhanced T1 weighted scanning showed a homogenously distinct lesion. The lesion is isointense to the spinal cord on T1 weighted images. It was iso-to-minimally hyperintense on T2 weighted images.

**Fig. 2:**
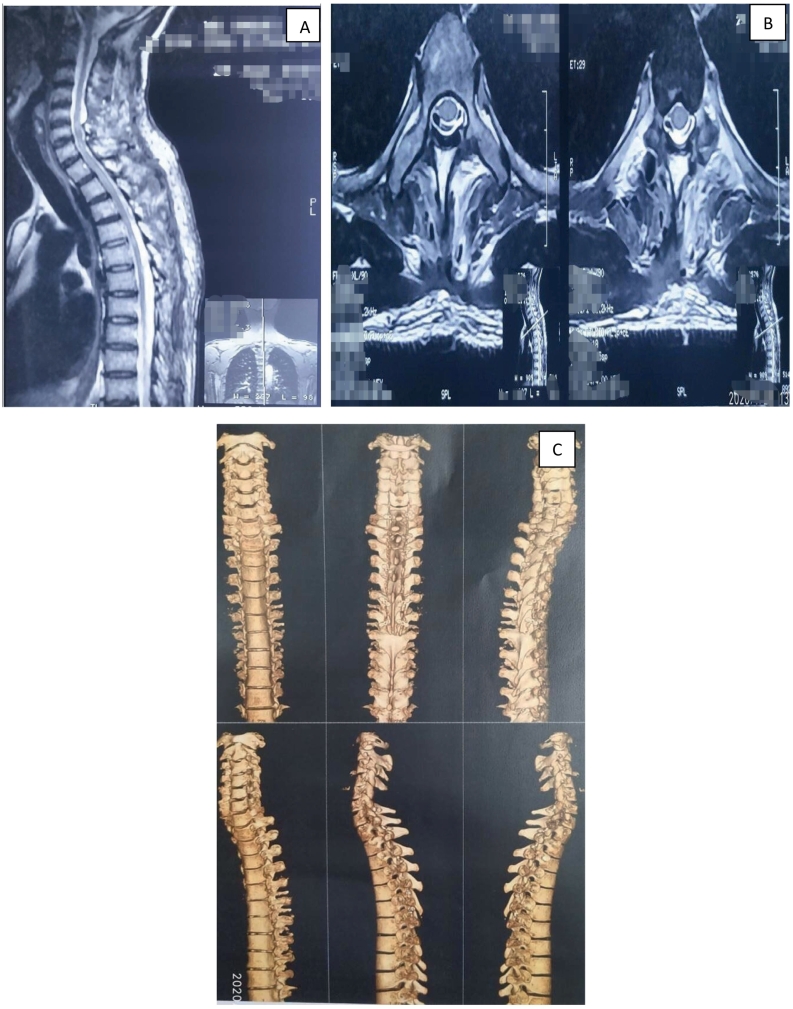
A- Postoperative T2 saggital MRI scan; B- Postoperative T2 axial MRI scan; C- 3D CT scan showing laminoplasty.

## 5 Discussion

Rosai-Dorfman Disease (RDD) is a self-limiting, benign illness with no known cause. It's marked by an overabundance of histiocytes in the body's lymph nodes, with involvement of the most common cervical lymph node [2]. However, extranodal disorders are rare, with <5%. The clinical presentation is usually bilateral, cervical lymphadenopathy of varying sizes and degrees with other systemic symptoms including low-grade fever, malaise, weight loss, anemia [3]. In our case, we encountered what we believe is a case of one of the most extended segment isolated spinal Rosai-Dorfman Disease, with the patient presenting progressive neurological (sensory and motor) functional loss of the lower limbs as well urinary retention.

Radiologically, the patient has a spinal tumor extending from C4-D6 levels. The differential diagnoses of this spinal involvement with the associated clinical features include reactive sinus histiocytosis, malignant histiocytosis, hemophagocytic syndrome, tuberculosis (Pott's disease), and lymphoma [8].

On histopathological assessment, the findings of clinical features such as emperipolesis (histocytes containing intact lymphocytes), atypia of histocytes, and presence of polyclonal plasma cells and lymphocytes suggested the diagnosis to be a lymphoproliferative disorder. Immunocytohistochemistry showed histocytes positive for S100, CD 68, and negative for CD30, which aided in reaching the diagnosis as RDD.

There is no specific laboratory test to diagnose RDD; similarly, in our case, laboratory investigations revealed no significant findings on CBC, CRP, serum glucose, serum creatinine tests, and serological tests were negative for hepatitis B, hepatitis C, and HIV, and blood and sputum culture were also harmful to Klebsiella and Brucella.

Haemophagocytic syndrome, Langerhans cell histiocytosis, reactive lymph node hyperplasia, and lymphoma are cytological diagnoses. Histiocytes phagocytose red cells in the haemophagocytic syndrome, and Langerhans cell histiocytes feature grooved and twisted nuclei with cd1a positive. Reactive lymphadenopathy lacks emperipolesis, and immunohistochemistry reveals negative S-100, but Reed-Sternberg cells are typical of Hodgkin lymphoma.

RDD's pathophysiology is unknown, while numerous experts have suggested it is linked to infection and immunodeficiency. Epstein-Barr virus, parvovirus B19, herpes virus type 6, polyomavirus, Klebsiella, Brucella, and cytomegalovirus have been suggested as possible causes but have yet to be proven. The other pathway is immune dysfunction or an abnormally amplified immune response to an infectious pathogen or antigen that induces histiocyte proliferation.

The pathophysiology of RDD is unknown, and whether it should be categorized as a neoplastic or benign condition is debatable. RDD cells were discovered to be polyclonal in previous research. However, recent reports of MAP-ERK pathway changes in around a third of RDD patients imply that at least a subset of these people may be cancerous. An RDD/LCH overlap has also been described in the past. Combined with the growing body of molecular and clinical evidence, these findings support the hypothesis that a subset of RDD is neoplastic and connected to the other histiocytic neoplasms [9].

Surgical excision and laminoplasty have proven to be a practical course of treatment as our patient showed remarkable improvement of neurological function. Medical intervention, such as a combination of alkylating drugs and vinca alkaloid with prednisone, which was also employed in our treatment protocol (CVP regimen), has also shown promise.

## 6 Conclusion

Extranodal sinus histiocytosis, particularly isolated spinal RDD, is an infrequent entity. The disease manifestations can suggest a wide range of differential diagnoses, rendering it a challenging case to approach. Histological examinations and immunophenotyping are used to make the diagnosis. Surgical resection is a practical treatment choice to relieve spinal cord compression, as we have shown in our case, which to our knowledge, has the most extended spinal segment involvement by spinal Rosai-Dorfman disease. Further researches on its origin, onset, and possible adjuvant therapies would make this disease a notable differential diagnosis of dural-based lesions in the neurosurgical field.

## Sources of funding

Non declared.

## Ethical approval

Hospital exempts ethics approval for reported cases.

## Consent

Written informed consent was obtained from the patient for publication of this case report and accompanying images. A copy of the written consent is available for review by the Editor-in-Chief of this journal on request.

## CRediT authorship contribution statement

All authors equally contributed to the analysis and writing of the manuscript.

## Research registration

Not applicable.

## Guarantor

Md Moshiur Rahman, Assistant Professor, Neurosurgery Department, Holy Family Red Crescent Medical College, Dhaka, Bangladesh, Email: dr.tutul@yahoo.com.

## Declaration of competing interest

The authors declare that they have no known competing financial interests or personal relationships that could have appeared to influence the work reported in this paper.
